# Stable Yet Destabilised: Towards Understanding Brain Network Dynamics in Psychogenic Disorders

**DOI:** 10.3390/jcm14030666

**Published:** 2025-01-21

**Authors:** Mostafa Badr, Timo Bröhl, Nayrin Dissouky, Christoph Helmstaedter, Klaus Lehnertz

**Affiliations:** 1 Department of Epileptology, University of Bonn Medical Center, Venusberg Campus 1, 53127 Bonn, Germany; mostafa.badr@ukbonn.de (M.B.); timo.broehl@uni-bonn.de (T.B.); nayrin.dissouky@ukbonn.de (N.D.);; 2 Helmholtz-Institute for Radiation and Nuclear Physics, University of Bonn, Nussallee 14–16, 53115 Bonn, Germany; 3 Interdisciplinary Center for Complex Systems, University of Bonn, Brühler Straße 7, 53175 Bonn, Germany

**Keywords:** epilepsy, psychogenic non-epileptic seizures, functional neurological disorders, psychopathology, EEG, complex networks, time-evolving functional brain networks, robustness, stability, destabilization

## Abstract

**Background:** Psychogenic non-epileptic seizures (PNES) are seizure-like episodes that resemble behavioral aspects observed for epileptic seizures but are without the abnormal electrical activity typically seen in epilepsy. The lack of an etiologic model for PNES as well as limitations of available diagnostic methods largely hinders a clear-cut distinction from epilepsy and from a normal functioning brain. **Methods:** In this study, we investigate the brain dynamics of people with PNES and people with epilepsy during phases far-off seizures and seizure-like events as well as the brain dynamics of a control group. Probing for differences between these groups, we utilise the network ansatz and explore local and global characteristics of time-evolving functional brain networks. We observe subject-specific differences in local network characteristics across the groups, highlighting the physiological functioning of specific brain regions. Furthermore, we observe significant differences in global network characteristics—relating to communication, robustness, and stability aspects of the brain. **Conclusions:** Our findings may provide new insights into the mechanisms underlying PNES and offer a promising diagnostic approach to differentiate them from epilepsy.

## 1. Introduction

Psychogenic non-epileptic seizures (PNES) are affiliated with functional neurological disorders (FNDs) and constitute seizure-like behavioral phenomena [1]. PNES are characterised by paroxysmal alterations in behavior, awareness, and autonomic function [2,3]. They are most commonly diagnosed in early adulthood, but can affect children as young as five years old and older adults [4,5,6]. The prevalence of PNES is 4.9 cases per 100,000 individuals per year [7]. Several factors have been identified as influential in the development and maintenance of PNES, including trauma, familial dysfunction, stressful life events, poor interpersonal skills and affect regulation, somatisation, psychopathology, personality factors, and avoidant coping styles [8,9]. Accordingly, PNES is thought to originate within a stress-diathesis model [10,11]. It is connected to psychological factors and linked to pre-existing psychiatric conditions such as conversion disorders, dissociative disorders, and trauma-related conditions [12].

Unlike epileptic seizures, which are understood to be caused by abnormal brain activity, mostly based on brain pathology, PNES are seizure-like events characterised by a lack of typical ictal patterns evident in EEG recordings, with manifestations that are highly heterogeneous and variable. This has made it difficult to develop a universal etiologic model of the disease [12,13]. Since treatment of PNES and epilepsy is approached differently, it is important to accurately diagnose PNES and to differentiate it from epilepsy. Misdiagnosis can result in an administration of inappropriate or even harmful therapy. Consequently, people with epilepsy may be incorrectly not treated with anti-seizure medication (ASM), while those with PNES are [5,14]. Wrong treatment of people with PNES can cause unnecessary negative behavioral and or cognitive side effects, thereby exacerbating the disease condition, and this increases healthcare costs [15].

Traditional diagnostic tools for differentiating PNES from epilepsy include video electroencephalographic monitoring (vEEG) and characterising seizure semiology. However, the clinical representation of PNES and epileptic seizures are quite similar in many aspects, making differentiation based solely on clinical presentation challenging [12]. Both conditions, PNES and epilepsy, can present with convulsive movements, altered consciousness, and a wide range of motor, sensory, and emotional phenomena. This clinical overlap necessitates the use of additional diagnostic tools to ensure accurate differentiation between PNES and epileptic seizures [16,17]. The gold standard for diagnosis of PNES to date is still capturing a typical event on vEEG and showing the lack of epileptiform activity during the seizure, supplemented by a congruent medical history [2].

However, the apparent unpredictability of seizures or seizure-like events during EEG monitoring represents a significant challenge in clinical practice, where further EEG monitoring for an extended period of time is always necessary to detect these events and subsequently to determine the underlying etiology. Therefore, identifying disease-specific alterations in brain dynamics from the event-free phase—the interval between two successive seizures or seizure-like events—would be highly beneficial, as it would facilitate the differentiation between conditions without the need for prolonged EEG monitoring.

Over the last decade, epilepsy and FNDs like PNES [1] have been conceptualised as network disorders [18,19,20], adopting the consideration of the brain as a network, that is a functionally and anatomically connected, bilaterally represented, set of cortical and subcortical brain structures and regions in which activity in any one part affects activity in all the others [21].

Despite significant advancements in EEG-based functional brain network studies of neurological disorders [18,19], and particularly of epilepsy [22,23,24,25], characteristics of time-evolving functional brain networks during the event-free phase in people with PNES [26,27] remain largely underexplored. These networks consist of vertices that represent the sampled brain regions and of edges that represent interactions between the dynamics of these regions, with the latter being characterised with suitable time-series-analysis techniques. Disease-specific alterations of the functional networks can be assessed by utilizing graph-theoretical metrics that evaluate network characteristics from the global to the local scale. On the global scale, metrics assessing a network’s segregation (clustering coefficient), integration (average shortest path length), efficiency to spread information (diameter), robustness (assortativity), and stability (synchronizability) have identified disease-specific modifications. Conversely, on the local scale, metrics (such as centralities) highlight subject-specific alterations and assess the (normal) physiological functioning of specific brain regions.

We hypothesise that the network approach—as described above—can help to improve the characterisation of brain dynamics during the event-free phase in PNES, which could ultimately lead to an improvement of its clinical diagnosis.

## 2. Materials and Methods

### 2.1. Subjects

Subjects admitted to the Department of Epileptology at the University Hospital Bonn between January 2020 and March 2024 were screened for eligibility for this study. After applying the specified inclusion and exclusion criteria (detailed below), data from 85 subjects were included in the final analysis. The subjects had signed informed consent that the clinical data might be used and published for research purposes. The study protocol had been approved by the ethics committee of the University of Bonn and is in accordance with the tenets of the Declaration of Helsinki. All experiments were performed in accordance with relevant guidelines and regulations.

### 2.2. Deriving Time-Evolving Functional Brain Networks from EEG Recordings

We selected 1-hour excerpts from continuous multiday EEG recordings according to the following criteria: (i) subjects were in a relaxed state of wakefulness; (ii) there was neither alteration in CNS medication (if taking any) nor the application of activation methods (such as photic stimulation, hyperventilation, or sleep deprivation) during the preceding 24 h; (iii) no seizures or seizure-like behavioral phenomena occurred during the preceding 24 h; (iv) data were recorded during the mornings to minimise possible influences from biological rhythms [28]; (v) data are free from strong artifacts such as subject movements or amplifier saturation.

EEG data were recorded with 19 electrodes (international 10–20 EEG system, with Cz as the physical reference), and data acquisition was performed with a 256 Hz sampling rate using a 16 bit analogue-to-digital converter. EEG data were band-pass filtered offline between 1–45 Hz (4th-order Butterworth characteristic), and a notch filter (3rd-order) suppressed contributions at the line frequency (50 Hz).

Our time-evolving functional brain networks consist of vertices and time-dependent edges and are weighted and fully connected networks [25]. We associated vertices with brain regions sampled by the EEG electrodes. We derived time-dependent edges by estimating the strength of interactions (mean phase coherence [29]) between EEG signals at the vertices, regardless of their anatomical connections, using a (non-overlapping) sliding-window approach [30,31,32,33]. The duration of each window amounted to 20 s (5120 data points), which represents a compromise between the required statistical accuracy for the calculation of the mean phase coherence and approximate stationarity within a window length.

### 2.3. Characterising Evolving Functional Brain Networks on Global and Local Scales

For each snapshot network in the time-dependent sequence of functional brain networks, we assessed global and local characteristics relevant for the diagnosis of central nervous system disorders [19,23,34,35].

On the global network scale, we assessed three topological characteristics. The average clustering coefficient *C* characterises the network’s functional segregation; the lower the *C* is, the more segregated the weighted fully connected network is. The average shortest path length *L* characterises the network’s functional integration; the lower the *L* is, the more integrated the weighted fully connected network is. Functional segregation (integration) reflects independent (dependent) information processes between brain regions [36]. The network diameter *D* is associated with the length of the longest of the shortest paths in the network, determining how efficiently information can spread through a network [37].

Additionally, we assessed the network’s stability and robustness characteristics. Synchronisability *S* assesses the network’s vulnerability to get synchronised by an admissible input activation [38,39]: the higher the *S* is, the more easily the synchronised state can be perturbed. Assortativity *A* captures the tendency of network edges to connect vertices with similar or equal properties [40]. If edges preferentially connect vertices with dissimilar properties, such a network is called disassortative. Disassortative networks are more vulnerable to perturbations and appear to be easier to synchronise than assortative ones.

On the local network scale, we assessed the importance of vertices and edges by employing four different centrality metrics, jointly defined for vertices and edges [41,42]. We differentiate between strength-based concepts (strength/nearest-neighbor centrality $C^{S}$/$C^{N}$ and eigenvector centrality $C^{E}$) and path-based concepts (closeness centrality $C^{C}$ and betweenness centrality $C^{B}$). Within each group, the former concepts are more sensitive to local aspects of the network, while the latter concepts are more sensitive to global aspects. Both groups provide non-redundant information about the role single constituents play in the larger network. Constituents with high values of strength-based centralities ($C^{S}$, $C^{N}$, and $C^{E}$) are thought to affect (and to be affected by) the rest of the network more strongly than constituents with smaller values. Constituents with high values of path-based centralities ($C^{C}$ and $C^{B}$) are important for information transport phenomena on a network, e.g., being bridges connecting remote network regions ($C^{B}$ high) or reaching other constituents via shortest paths ($C^{C}$ high).

### 2.4. Statistical Analyses

Differences in the temporal medians of the global network characteristics between the three subject groups were assessed using the Mann–Whitney U-Test (p<0.05, after Bonferroni correction).

Analysis and code implementation has been done with Python 3.12.7. Figures have been made in Python 3.12.7 and Inkscape.

## 3. Results

We investigated characteristics of time-evolving functional brain networks in a sizable cohort of people with PNES and those with epilepsy, as well as in comparison to the normal brain dynamics of a control group. Data from 85 subjects (42 females, 43 males; ages: 19–88 yrs; median age: 33 yrs) were included in the final analysis after exclusions:

- A total of 30 individuals without any history of neurological disorders (12 females, 18 males; age range: 19–88 yrs; median age: 37 yrs; control group G1);
- A total of 22 individuals with psychogenic non-epileptic seizures (11 females, 11 males; age range: 21–82 yrs; median age: 29 yrs; group G2);
- A total of 33 individuals with focal epilepsy (19 females, 14 males; age range: 19–81 yrs; median age: 35 yrs; group G3).

Among those individuals with epilepsy, 16 had a left-hemispheric seizure origin, while 8 had a right-hemispheric origin. The other nine individuals with epilepsy either had an unclear or a bi-temporal seizure origin.

### 3.1. Global Characteristics of Time-Evolving Functional Brain Networks

**Figure 1:**
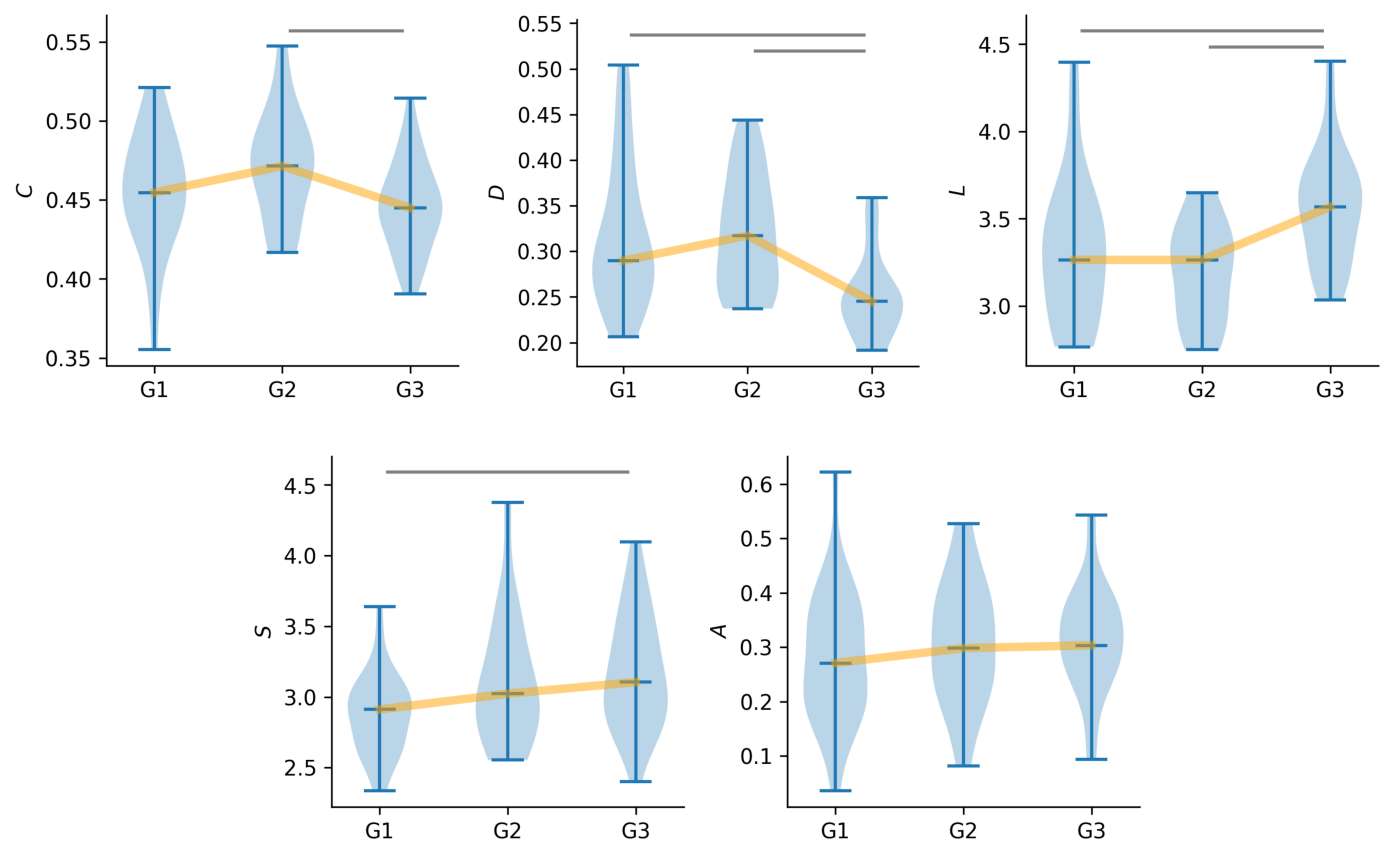
Global network characteristics differ between groups. Sample distributions of average clustering coefficient *C*, diameter *D*, average shortest path length *L*, synchronizability *S*, and assortativity *A*. G1 = control group, G2 = PNES group, G3 = epilepsy group). Yellow lines connect median values and are for eye guidance only. Grey vertical lines indicate significant differences between groups.

On the global network scale, we observe clear cut and even significant distinctions in specific characteristics between the three groups (Figure 1). Surprisingly, these characteristics observed for people with PNES (G2) not only contrast with the characteristics observed in the control group (G1) but further those observed for people with epilepsy (G3). Significant differences between groups G2 and G3 are evident and observed for the three investigated topological characteristics, i.e., clustering coefficient (*C*), diameter (*D*), and average shortest path length (*L*). This points to overall more integrated (smaller *L*) and less segregated (higher *C*) functional networks, with a generally more efficient flow of information (larger *D*), in group G2 compared to group G3. While the networks of groups G1 and G2 appear to be comparably segregated (no significant differences in *C*; comparable median values), the overall integration and flow of information in the networks of groups G1 and G3 differ significantly. In the networks of the latter group, the flow of information (*D*) is lower than that observed in the networks of the control group. Conversely, the network integration (*L*) of groups G1 and G2 is comparable, while the network integration of G3 is stronger, on average.

Despite these prominent differences observed in network integration and segregation across the three groups, aspects of the networks’ robustness (assortativity *A*) remain consistent. In aspects of the networks’ stability (synchronizability *S*), those of group G2 appear to be, on average (median values), slightly less stable than those of group G1 but more stable than the networks of group G3. Nevertheless, differences observed with *S* were only significant when comparing the networks of groups G1 and G2, pointing to a gradual decrease in the networks’ stability from the control group over people with PNES to people with epilepsy.

### 3.2. Local Characteristics of Time-Evolving Functional Brain Networks

**Figure 2:**
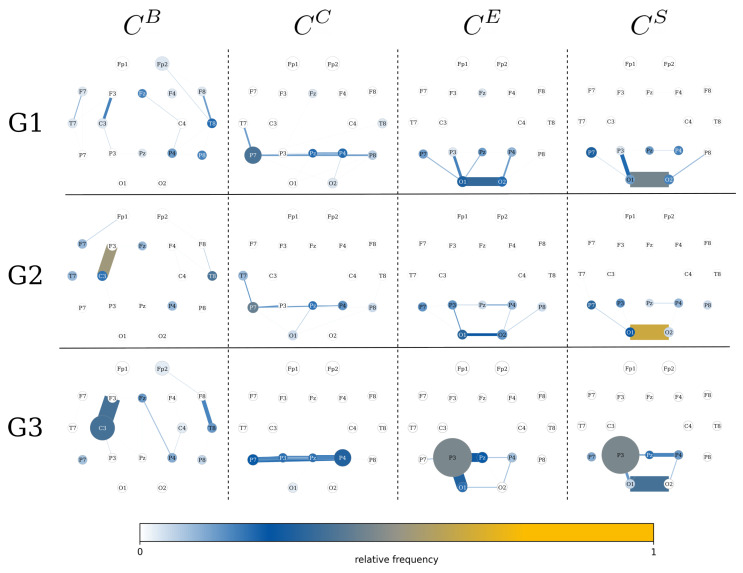
Local network characteristics differ between groups. The duration of network vertices and edges that are the most important is encoded in their size: the larger/thicker they are, the longer they were most important. Color encodes the relative frequency of subjects for which a network constituent was the most important at any time. Importance is estimated with betweenness centrality $C^{B}$, closeness centrality $C^{C}$, eigenvector centrality $C^{E}$, and strength/nearest-neighbor centrality $C^{S}$/$C^{N}$. Networks are depicted in the layout of the 10–20 EEG system [44]. G1 = control group, G2 = PNES group, G3 = epilepsy group).

On the local network scale (Figure 2), different network constituents are deemed important based on employed centrality metrics, which aligns with previous research [34,35,43]. Here, we examine the total amount of time a constituent is considered the most important. We also consider the proportion of subjects for which a specific constituent is considered the most important at any time.

With closeness centrality $C^{C}$, we observe a distinct substructure in (temporo)-centro-parietal brain regions over both hemispheres across the networks of all groups. In the networks of the control group and in terms of specific brain regions, this substructure highlights a left-hemispheric parietal dominance (vertex P7), while the importance of interactions between brain regions appears to be more diffusely distributed along the axis from the left to the right hemisphere (vertex P7 to vertex P8). In group G2, the proportion of subjects is even larger, for which the left-parietal brain region (vertex P7) is deemed most important—the same observation holds in comparison to group G3. Also, for the networks of group G2, the described temporo-centro-parietal substructure is more retracted to the left brain hemisphere compared to the networks of group G1. This is even more pronounced for the networks of group G3, yet contrarily highlighting the right parietal brain region as most important (around vertex P4). This overall points to a gradual retraction and more pronounced centering of the described substructure in the (temporo)-centro-parietal brain region towards a more centro-parietal brain region with a slight right-hemispheric dominance observable for the networks from the control group (G1) to people with PNES (G2) to people with epilepsy (G3).

In the case of the strength-based centrality concepts ($C^{S}$/$C^{N}$ and $C^{E}$), we further observe a distinct substructure in the networks across all three groups, and while it is similar to the previously described one, it further and more dominantly spans into the occipital brain regions (vertices O1 and O2). A distinction between these substructures observed in groups G1 and G2 is only limitedly possible, yet the networks of group G3 show a very pronounced highlighting of the left-hemispheric parietal brain region (vertex P3), both in terms of the duration for which it was most important and the proportion of subjects for which this specific brain region is deemed most important. Nevertheless and specifically when considering eigenvector centrality $C^{E}$, people with PNES are accentuated compared to people from the other groups, as the communication between left- and right-hemispheric occipital brain regions is observed in the majority of subjects with PNES.

The time-evolving functional brain networks of groups G1 and G2 turned out to be similar in their characteristics. This suggests that while PNES manifests as seizure-like episodes, its underlying functional network organization is closer to that of the control group than to that of people with epilepsy. This described distinction between the networks of people with PNES and those of people with epilepsy emphasises the less focal and more diffused involvement of network constituents in people with PNES, possibly reflecting its psychogenic origin.

## 4. Discussion

We identified distinct differences in global and local characteristics of time-evolving functional brain networks from the event-free phase, displaying a partly gradual change from the control group to people with PNES to those with epilepsy. Regarding global network characteristics, our study identified higher network integration and lower network segregation in people with PNES compared to people with epilepsy, indicating more diffused network dynamics in people with PNES. This novel finding may support the hypothesis that PNES may be precipitated by a global psychological stress response, frequently linked to substantial psychosocial stressors [45,46]. This widespread, diffused network activity may be concomitant with emotional dysregulation and dissociative mechanisms, which are commonly observed in people with PNES [12,20,47]. In contrast, the higher network segregation and lower network integration observed in people with epilepsy during the event-free phase compared to those with PNES reflects fewer diffused network dynamcis. This may be due to the focal nature of epileptic disorders in our cohort [35,48].

As anticipated, the control group in our cohort demonstrated intermediate levels of network segregation and network integration [35]. This suggests a more balanced and typical brain network organization compared to the extreme patterns observed in both people with PNES and those with epilepsy during the event-free phase. This intermediate network configuration reflects optimal brain dynamics, wherein segregation and integration are precisely regulated to facilitate efficient communication between specialised regions.

The brain networks of people with epilepsy demonstrated the lowest stability, which is consistent with expectations based on the focal nature of epileptic dysfunction. This might be linked to challenges in sustaining effective communication between brain regions. Conversely, the brain networks of people with PNES during the event-free phase exhibited greater stability than those of people with epilepsy, though they remained less stable than those of the control group. This suggests that the underlying mechanisms involved in PNES exert a lesser extent of destabilization on functional brain networks in comparison to neurological disturbances typically observed in many people with epilepsy [49]. This may be attributed to epileptic dysfunction itself or, in some cases, to structural abnormalities [50,51,52].

Despite the marked variations in network integration, network segregation, and network stability observed across the three groups in our cohort, network robustness remained consistent. This suggests that, across all conditions, the brain displays a fundamental tendency for analogous regions to connect. Even in the disorganised networks of PNES and epilepsy, the fundamental structural framework that maintains connectedness between analogous vertices remains intact. This robustness suggests that, despite pathophysiological differences, the core organizational properties of the brain are preserved to some extent [53].

Regarding local network characteristics, our findings are in general accordance with those made in regard to global network characteristics. While strength-based centrality concepts (strength/nearest-neighbor centrality $C^{S}$/$C^{N}$ and eigenvector centrality $C^{E}$) indicate that the time-evolving functional brain networks of people with PNES are only limited differentiable from those of the control group during the event-free phases, the brain networks of people with epilepsy are clearly different. Yet, in people with epilepsy, left-hemispheric centro-parietal brain regions as well as the communication between these and left-hemispheric occipital brain regions are highlighted and more prominent over time. This may be attributed, at least in part, to the fact that the majority of people with epilepsy in our cohort have a left-hemispheric seizure origin.

Conversely, for path-based centrality concepts (closeness centrality $C^{C}$ and betweenness centrality $C^{B}$), a gradual change in the importance of specific network constituents was observed. While generally left-hemispheric fronto-central brain regions and communications between them are highlighted as most important, the duration of the respective network constituents being most important increased from the control group over people with PNES to people with epilepsy. Yet, especially the communication between these brain regions is more prevalent in people with PNES compared to people with epilepsy or the control group. Especially with closeness centrality $C^{C}$, parietal inter-hemispheric regions and the communications between them are highlighted. While this communication-axis (a substructure in the time-evolving functional brain network) in networks of the control group showed a left-hemispheric dominance, people with PNES showed a more diffusely distributed importance along this axis. Finally, for people with epilepsy, the described dominance showed a retraction towards the left hemisphere, and an overall gradual change from the left to the right brain hemisphere, from the control group to people with PNES to people with epilepsy. Together with prior research [54], these observations point to vital and fundamental subnetworks within the time-evolving functional networks, which can be associated with the concept of a resting-state network [55]. Overall, while PNES manifests as seizure-like episodes, its underlying local brain network organization is more closely aligned with that of the control group than that of people with focal epilepsy.

A limitation of our study is that our epilepsy cohort consisted solely of individuals with focal epilepsy. Given the differences in both network segregation and integration between focal and generalised epilepsy [35,56], as well as the resemblance of PNES semiology to generalised seizures, including patients with generalised epilepsy in future studies could further enhance the interpretability and clinical applicability of our findings. Moreover, network characteristics in people with PNES may be influenced by the frequency of episodes [57] while antiseizure medication may affect those in people with epilepsy [58]. These are important factors to consider in future studies.

## 5. Conclusions

To conclude, significant differences in global characteristics of time-evolving functional brain networks during the event-free phase across all groups were observed in our cohort, while local network characteristics were found to be largely similar in people with PNES, those with epilepsy, and the control group. A peculiar network de-stability observed for people with PNES places them in between normal functioning and epileptic brains. These findings may prove to be a valuable additional tool in understanding brain network dynamics in psychogenic disorders as well as in differentiating between PNES and epilepsy in clinical practice.

## Data Availability

The data that support the findings of this study are available from the corresponding author upon reasonable request. The data are not publicly available as they contain information that could compromise the privacy of research participants.

## References

[1] Fobian A.D., Elliott L. (2019). A review of functional neurological symptom disorder etiology and the integrated etiological summary model. J. Psychiatry Neurosci..

[2] Perez D.L., LaFrance W.C. (2016). Nonepileptic seizures: An updated review. CNS Spectr..

[3] Takasaki K., Stransky A.D., Miller G. (2016). Psychogenic nonepileptic seizures: Diagnosis, management, and bioethics. Pediatr. Neurol..

[4] Reuber M., Fernandez G., Bauer J., Helmstaedter C. (2002). Delays in the diagnosis of psychogenic non-epileptic seizures. (ABN Abstracts). J. Neurol. Neurosurg. Psychiatry.

[5] Reuber M., Fernández G., Bauer J., Helmstaedter C., Elger C.E. (2002). Diagnostic delay in psychogenic nonepileptic seizures. Neurology.

[6] Radmanesh M., Jalili M., Kozlowska K. (2020). Activation of functional brain networks in children with psychogenic non-epileptic seizures. Front. Hum. Neurosci..

[7] Duncan R., Razvi S., Mulhern S. (2011). Newly presenting psychogenic nonepileptic seizures: Incidence, population characteristics, and early outcome from a prospective audit of a first seizure clinic. Epilepsy Behav..

[8] Alsaadi T.M., Marquez A.V. (2005). Psychogenic nonepileptic seizures. Am. Fam. Physician.

[9] Bodde N., Brooks J., Baker G., Boon P., Hendriksen J., Mulder O., Aldenkamp A. (2009). Psychogenic non-epileptic seizures—Definition, etiology, treatment and prognostic issues: A critical review. Seizure.

[10] Keynejad R.C., Frodl T., Kanaan R., Pariante C., Reuber M., Nicholson T.R. (2019). Stress and functional neurological disorders: Mechanistic insights. J. Neurol. Neurosurg. Psychiatry.

[11] Weber S., Bühler J., Vanini G., Loukas S., Bruckmaier R., Aybek S. (2023). Identification of biopsychological trait markers in functional neurological disorders. Brain.

[12] Brown R.J., Reuber M. (2016). Towards an integrative theory of psychogenic non-epileptic seizures (PNES). Clin. Psychol. Rev..

[13] Hopp J.L. (2019). Nonepileptic episodic events. Contin. Lifelong Learn. Neurol..

[14] Gedzelman E.R., LaRoche S.M. (2014). Long-term video EEG monitoring for diagnosis of psychogenic nonepileptic seizures. Neuropsychiatr. Dis. Treat..

[15] Martin R.C., Gilliam F.G., Kilgore M., Faught E., Kuzniecky R. (1998). Improved health care resource utilization following video-EEG-confirmed diagnosis of nonepileptic psychogenic seizures. Seizure.

[16] Brigo F., Igwe S.C., Erro R., Bongiovanni L.G., Marangi A., Nardone R., Tinazzi M., Trinka E. (2015). Postictal serum creatine kinase for the differential diagnosis of epileptic seizures and psychogenic non-epileptic seizures: A systematic review. J. Neurol..

[17] Gledhill J.M., Brand E.J., Pollard J.R., St. Clair R.D., Wallach T.M., Crino P.B. (2021). Association of epileptic and nonepileptic seizures and changes in circulating plasma proteins linked to neuroinflammation. Neurology.

[18] Bullmore E., Sporns O. (2009). Complex brain networks: Graph theoretical analysis of structural and functional systems. Nat. Rev. Neurosci..

[19] Stam C.J. (2014). Modern network science of neurological disorders. Nat. Rev. Neurosci..

[20] Szaflarski J.P., LaFrance W.C. (2018). Psychogenic nonepileptic seizures (PNES) as a network disorder–evidence from neuroimaging of functional (psychogenic) neurological disorders. Epilepsy Curr..

[21] Spencer S. (2002). Neural networks in human epilepsy: Evidence of and implications for treatment. Epilepsia.

[22] Richardson M. (2010). Current themes in neuroimaging of epilepsy: Brain networks, dynamic phenomena, and clinical relevance. Clin. Neurophysiol..

[23] Lehnertz K., Ansmann G., Bialonski S., Dickten H., Geier C., Porz S. (2014). Evolving networks in the human epileptic brain. Physica D.

[24] Lehnertz K., Bröhl T., von Wrede R. (2023). Epileptic-network-based prediction and control of seizures in humans. Neurobiol. Dis..

[25] Bröhl T., Rings T., Pukropski J., von Wrede R., Lehnertz K. (2024). The time-evolving epileptic brain network: Concepts, definitions, accomplishments, perspectives. Front. Netw. Physiol..

[26] Barzegaran E., Joudaki A., Jalili M., Rossetti A.O., Frackowiak R.S., Knyazeva M.G. (2012). Properties of functional brain networks correlate with frequency of psychogenic non-epileptic seizures. Front. Hum. Neurosci..

[27] Ahmadi N., Pei Y., Carrette E., Aldenkamp A.P., Pechenizkiy M. (2020). EEG-based classification of epilepsy and PNES: EEG microstate and functional brain network features. Brain Inform..

[28] Lehnertz K., Rings T., Bröhl T. (2021). Time in Brain: How Biological Rhythms Impact on EEG Signals and on EEG-Derived Brain Networks. Front. Netw. Physiol..

[29] Mormann F., Lehnertz K., David P., Elger C.E. (2000). Mean phase coherence as a measure for phase synchronization and its application to the EEG of epilepsy patients. Physica D.

[30] Kuhnert M.T., Elger C.E., Lehnertz K. (2010). Long-term variability of global statistical properties of epileptic brain networks. Chaos.

[31] Dickten H., Porz S., Elger C.E., Lehnertz K. (2016). Weighted and directed interactions in evolving large-scale epileptic brain networks. Sci. Rep..

[32] Geier C., Lehnertz K. (2017). Long-term variability of importance of brain regions in evolving epileptic brain networks. Chaos.

[33] von Wrede R., Bröhl T., Rings T., Pukropski J., Helmstaedter C., Lehnertz K. (2022). Modifications of Functional Human Brain Networks by Transcutaneous Auricular Vagus Nerve Stimulation: Impact of Time of Day. Brain Sci..

[34] Rings T., von Wrede R., Bröhl T., Schach S., Helmstaedter C., Lehnertz K. (2021). Impact of transcutaneous auricular vagus nerve stimulation on large-scale functional brain networks: From local to global. Front. Physiol..

[35] von Wrede R., Rings T., Bröhl T., Pukropski J., Schach S., Helmstaedter C., Lehnertz K. (2022). Transcutaneous Auricular Vagus Nerve Stimulation Differently Modifies Functional Brain Networks of Subjects With Different Epilepsy Types. Front. Hum. Neurosci..

[36] Tononi G., Sporns O., Edelman G.M. (1994). A measure for brain complexity: Relating functional segregation and integration in the nervous system. Proc. Natl. Acad. Sci. USA.

[37] Newman M. (2018). Networks.

[38] Pecora L.M., Carroll T.L. (1998). Master stability functions for synchronized coupled systems. Phys. Rev. Lett..

[39] Barahona M., Pecora L.M. (2002). Synchronization in small-world systems. Phys. Rev. Lett..

[40] Newman M.E.J. (2003). The structure and function of complex networks. SIAM Rev..

[41] Bröhl T., Lehnertz K. (2019). Centrality-based identification of important edges in complex networks. Chaos.

[42] Bröhl T., Lehnertz K. (2022). A straightforward edge centrality concept derived from generalizing degree and strength. Sci. Rep..

[43] Lehnertz H., Broehl T., Rings T., Von Wrede R., Lehnertz K. (2023). Modifying functional brain networks in focal epilepsy by manual visceral-osteopathic stimulation of the vagus nerve at the abdomen. Front. Netw. Physiol..

[44] Seeck M., Koessler L., Bast T., Leijten F., Michel C., Baumgartner C., He B., Beniczky S. (2017). The standardized EEG electrode array of the IFCN. Clin. Neurophysiol..

[45] Reuber M., Jamnadas-Khoda J., Broadhurst M., Grunewald R., Howell S., Koepp M., Sisodiya S., Walker M. (2011). Psychogenic nonepileptic seizure manifestations reported by patients and witnesses. Epilepsia.

[46] LaFrance W.C., Devinsky O. (2002). Treatment of nonepileptic seizures. Epilepsy Behav..

[47] van der Kruijs S.J., Bodde N.M., Vaessen M.J., Lazeron R.H., Vonck K., Boon P., Hofman P.A., Backes W.H., Aldenkamp A.P., Jansen J.F. (2012). Functional connectivity of dissociation in patients with psychogenic non-epileptic seizures. J. Neurol. Neurosurg. Psychiatry.

[48] Jiruska P., de Curtis M., Jefferys J.G.R., Schevon C.A., Schiff S.J., Schindler K. (2013). Synchronization and desynchronization in epilepsy: Controversies and hypotheses. J. Physiol..

[49] Elger C.E., Helmstaedter C., Kurthen M. (2004). Chronic epilepsy and cognition. Lancet Neurol..

[50] Engel J., Thompson P.M., Stern J.M., Staba R.J., Bragin A., Mody I. (2013). Connectomics and epilepsy. Curr. Opin. Neurol..

[51] Bernhardt B.C., Bonilha L., Gross D.W. (2015). Network analysis for a network disorder: The emerging role of graph theory in the study of epilepsy. Epilepsy Behav..

[52] Sporns O., Bassett D.S. (2018). New trends in connectomics. Netw. Neurosci..

[53] Bullmore E., Sporns O. (2012). The economy of brain network organization. Nat. Rev. Neurosci..

[54] Bröhl T., Von Wrede R., Lehnertz K. (2023). Impact of biological rhythms on the importance hierarchy of constituents in time-dependent functional brain networks. Front. Netw. Physiol..

[55] Raichle M.E. (2015). The brain’s default mode network. Annu. Rev. Neurosci..

[56] Chowdhury F.A., Woldman W., FitzGerald T.H., Elwes R.D., Nashef L., Terry J.R., Richardson M.P. (2014). Revealing a brain network endophenotype in families with idiopathic generalised epilepsy. PLoS ONE.

[57] Barzegaran E., Carmeli C., Rossetti A.O., Frackowiak R.S., Knyazeva M.G. (2016). Weakened functional connectivity in patients with psychogenic non-epileptic seizures (PNES) converges on basal ganglia. J. Neurol. Neurosurg. Psychiatry.

[58] Höller Y., Helmstaedter C., Lehnertz K. (2018). Quantitative pharmaco-electroencephalography in antiepileptic drug research. CNS Drugs.

